# Antioxidants Mediate Both Iron Homeostasis and Oxidative Stress

**DOI:** 10.3390/nu9070671

**Published:** 2017-06-28

**Authors:** Mustapha Umar Imam, Shenshen Zhang, Jifei Ma, Hao Wang, Fudi Wang

**Affiliations:** 1 Department of Nutrition, Precision Nutrition Innovation Center, School of Public Health, Zhengzhou University, Zhengzhou 450001, China; mustyimam@gmail.com (M.U.I.); zsslb2005@163.com (S.Z.); mjf15188351024@163.com (J.M.); 0016896@zju.edu.cn (H.W.); 2 Department of Nutrition, Nutrition Discovery Innovation Center, School of Public Health, Collaborative Innovation Center for Diagnosis and Treatment of Infectious Diseases, School of Medicine, Zhejiang University, Hangzhou 310058, China

**Keywords:** iron homeostasis, iron overload, antioxidants, plant extracts, oxidative stress, ferroptosis

## Abstract

Oxidative stress is a common denominator in the pathogenesis of many chronic diseases. Therefore, antioxidants are often used to protect cells and tissues and reverse oxidative damage. It is well known that iron metabolism underlies the dynamic interplay between oxidative stress and antioxidants in many pathophysiological processes. Both iron deficiency and iron overload can affect redox state, and these conditions can be restored to physiological conditions using iron supplementation and iron chelation, respectively. Similarly, the addition of antioxidants to these treatment regimens has been suggested as a viable therapeutic approach for attenuating tissue damage induced by oxidative stress. Notably, many bioactive plant-derived compounds have been shown to regulate both iron metabolism and redox state, possibly through interactive mechanisms. This review summarizes our current understanding of these mechanisms and discusses compelling preclinical evidence that bioactive plant-derived compounds can be both safe and effective for managing both iron deficiency and iron overload conditions.

## 1. Overview of Iron Metabolism

### 1.1. Dietary Requirements for Iron and Iron Absorption, Transport, Storage, and Utilization

The processes underlying iron metabolism are as complex as they are perplexing. Given that iron is an essential trace element required for maintaining physiological homeostasis, it is both surprising and somewhat alarming that iron overload and iron deficiency are common conditions in the general population. Upon closer inspection, however, the underlying causes of these conditions are not difficult to understand. Dietary iron is the principal source of iron in the body; in addition, iron can be recycled from body stores, particularly the liver and senescent red blood cells (RBCs). Interestingly, despite its important role in maintaining plasma iron levels, iron excretion is less regulated than iron absorption [1,2]. Moreover, excessive iron levels can lead to toxicity and cell death via free radical formation and lipid peroxidation; therefore, iron homeostasis is tightly regulated [3,4,5]. Blood loss through menstruation and other routes, sloughed intestinal mucosal cells, and sweating help the body maintain normal plasma iron levels. Iron is used primarily as a component of heme in RBCs for oxygen transport, while relatively smaller amounts are present in muscle as heme myoglobin and in the liver as ferritin [1,2]. In most other tissues in the body, iron serves as a component of protein cofactors in the form of iron-sulfur (Fe-S) clusters and heme, which are involved in the electron transport chain to produce ATP [6,7]. In this process, ferrous iron (Fe^2+^) and ferric iron (Fe^3+^) serve as an electron donor and acceptor, respectively. In effect, iron plays an essential role in maintaining adequate stores of ATP during oxidative phosphorylation in the mitochondrial inner membrane [8].

In industrialized countries, the majority of the population has adequate iron stores, usually totaling approximately 4–5 g (representing approximately 38 and 50 mg iron/kg body weight for adult women and men, respectively). Approximately 2.5 g of this iron is present in hemoglobin for oxygen transport, and another 2 g is stored in the form of ferritin, largely in the bone marrow, liver, and spleen [9]. In the bone marrow, iron is used for hemoglobin formation. In the liver, iron serves as the principal iron reserve, and the reticuloendothelial cells in the spleen recycle iron form senescent RBCs. Finally, a relatively small amount of iron (approximately 400 mg) is present in cellular proteins such as myoglobin and cytochromes, and even less (approximately 3–4 mg) is in the circulation bound to transferrin [1,2]. 

**Figure 1:**
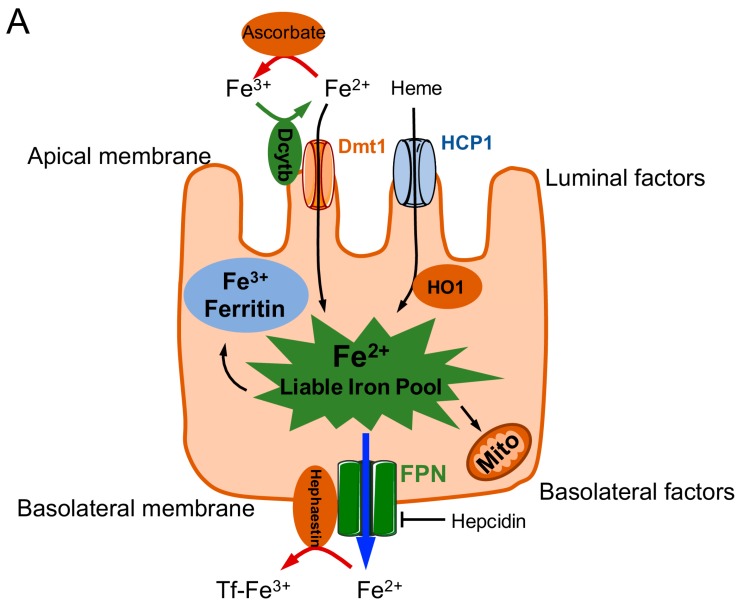

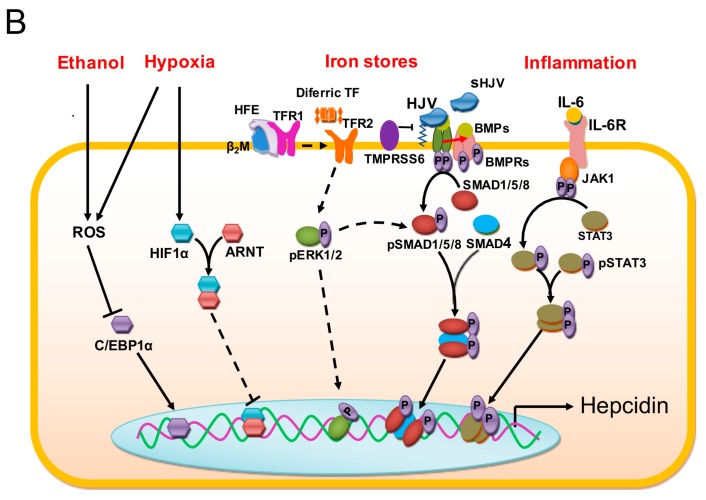
Schematic overview of iron transport into and across duodenal enterocytes (**A**) and the pathways involved in regulating transcription of the hepcidin antimicrobial peptide (*HAMP*) gene to drive hepcidin expression (**B**). Iron is imported as heme via the heme carrier protein 1 (HCP1) or as Fe^2+^ (after reduction by duodenal cytochrome b (Dcytb) via the divalent metal transporter (DMT) 1. The labile iron pool within the enterocyte can be stored as ferritin, utilized for mitochondrial oxidative phosphorylation, or exported via ferroportin (Fpn). Hephaestin or ceruloplasmin then converts Fe^2+^ to Fe^3+^, which then binds to transferrin (Tf). Hepcidin negatively regulates Fpn. ARNT: aryl hydrocarbon nuclear receptor translocator; β_2_m:beta-2-microglobulin; BMP: bone morphogenetic protein; BMPR: BMP receptor; C/EBP1α: CCAAT/enhancer-binding protein 1α; ERK1/2: extracellular signal-regulated kinase; HIF1α: hypoxia-inducible factor 1α; HFE: human hemochromatosis protein; HJV: hemojuvelin; HO1: heme oxygenase 1; IL-6: interleukin 6; IL-6R: IL-6 receptor; JAK: Janus kinase; Mito: mitochondria; p: phosphate group; ROS: reactive oxygen species; sHJV: serum HJV; SMAD1/5/8: mothers against decapentaplegic homolog1/5/8; SMAD4: mothers against decapentaplegic homolog 4; STAT3: signal transducer and activator of transcription 3; TfR: Tf receptor; TMPRSS6: transmembrane protease, serine 6.

Before iron can fulfill its crucial biological roles, it must be absorbed across intestinal enterocytes into the blood. Dietary iron from animal sources (blood and heme-containing proteins) is absorbed better than iron from plant sources (mitochondrial heme) [10,11]. Both heme-bound iron and ionic iron are absorbed at the apical surface of duodenal enterocytes (Figure 1). Ionic iron (Fe^3+^) is not bioavailable and must be reduced to Fe^2+^ by the duodenal enzyme cytochrome b reductase (Dcytb), a membrane-bound ferroxidase expressed at the duodenal brush border; Fe^2+^ can then be absorbed via divalent metal transporter 1 (DMT1), an enzyme that also transports other metals, including manganese, zinc, copper, and cobalt [1,2].

**Figure 2:**
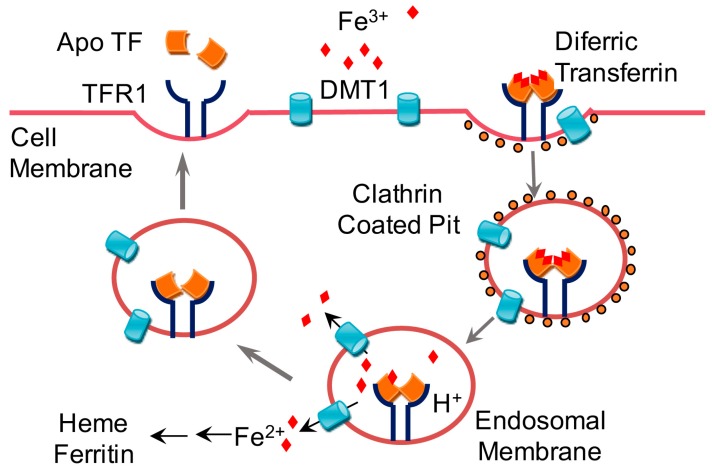
Schematic depiction of iron trafficking across the plasma membrane into the cell. Transferrin-(Tf-) bound iron is transported into the cell via transferrin receptor 1 (TfR1) or 2 (TfR2). The complex is endocytosed, and a decrease in luminal pH causes the release of iron from the complex. Tf that is completely free of iron (apotransferrin, ApoTf) and the TfR are then recycled back to the cell membrane. The labile pool of iron within the cell exits the endosome via divalent metal transporter 1 (DMT1) and is stored as ferritin. ApoTf: apotransferrin.

Once inside the enterocyte, iron is then stored in the cell as ferritin, where it is often lost as sloughed duodenal cells or is released into the body via ferroportin, after which it is oxidized back to Fe^3+^ in the basolateral side of the duodenum by the ferroxidase hephaestin (HEPH). Iron is then transported in the plasma as a complex with transferrin (Tf), which is detected by transferrin receptor 1 (TfR1) or 2 (TfR2) and endocytosed as the Fe-Tf complex [12] (Figure 2); TfR1-mediated endocytosis is approximately 30 times more efficient than TfR2-mediated endocytosis [13]. Alternatively, iron can be taken up via the Glyceraldehyde 3-phosphate dehydrogenase (GAPDH) pathway [14,15]. A decrease in pH within the resulting endosomes (due to of the active transport of protons into the endosomes) causes the complex to dissociate, after which transferrin and the TfR are recycled back to the cell membrane; transferrin is then available to bind iron again, and the TfR is again available for sensing new Fe-Tf complexes. The labile iron within the endosomes is transported to the cytoplasm via DMT1 after conversion to Fe^2+^ by a member of the Six transmembrane epithelial antigen of the prostate (STEAP) family of reductase proteins [16]. Conversely, extracellular iron that has been converted to Fe^2+^ state by a STEAP family reductase can enter the cell directly via an alternate surface transporter such as Zrt-Irt-like protein 14 (ZIP14, also known as SLC39A14), thereby contributing to the labile pool of iron [17]. This labile pool of iron is then stored as ferritin-bound or hemosiderin-bound iron [2,18]. Under steady-state conditions, the serum ferritin level is directly proportional to total body iron stores; therefore, serum ferritin is commonly used as a convenient laboratory test for estimating iron stores [19]. However, other pathological conditions unrelated to iron status—for example, chronic inflammation—have been shown to increase serum ferritin [16].

Ferritin is a globular protein complex consisting of 24 protein subunits; the primary function of ferritin is to store iron intracellularly in order to prevent iron toxicity [20,21]. Moreover, ferritin releases iron via a controlled mechanism in response to appropriate stimuli [22], thereby protecting against both iron deficiency and iron overload [1,2]. Extremely high amounts of ferritin—particularly within reticuloendothelial cells—are converted to hemosiderin, which is more difficult to access. When iron is released from cells such as neurons, erythrocytes, and macrophages, it is exported via ferroportin in the same manner described above, although ceruloplasmin and hephaestin can also oxidize iron to form Fe^3+^ in other cells outside of the duodenum [16,23].

### 1.2. Regulation of Iron Stores

In humans, iron homeostasis is coordinated by a series of complex, tightly regulated processes. At the systemic level, iron levels are regulated through the controlled absorption of dietary iron via enterocytes, duodenal cell sloughing, sweating, blood loss, and recycling of systemic iron [1,2]. At the cellular level, however, regulatory molecules such as ferroportin and hepcidin contribute to iron regulation [23,24]. Hepcidin (encoded by the hepcidin antimicrobial peptide, or *HAMP*, gene) is a peptide hormone produced primarily in the liver, and its expression is tightly controlled at the transcriptional level by iron levels, erythroferrone (erythropoiesis), inflammation, and hypoxia via the bone morphogenetic protein/suppressor of mothers against decapentaplegic (BMP/SMAD) and Janus kinase/signal transducer and activator of transcription (JAK/STAT) signaling pathways (Figure 1) [16,25]. Hepcidin can post-translationally repress ferroportin, whereas erythroferrone produced by erythroblasts inhibits ferroportin synthesis, leading to increased availability of iron for use in hemoglobin [26]. Hepcidin represses ferroportin by causing its internalization, thereby decreasing iron export. In addition, hepcidin can downregulate both TfR1 and DMT1, also reducing iron export [27]. Conversely, in response to iron deficiency, the body can synthesize additional Dcytb, DMT1, and ferroportin [16,24,25]. 

**Figure 3:**
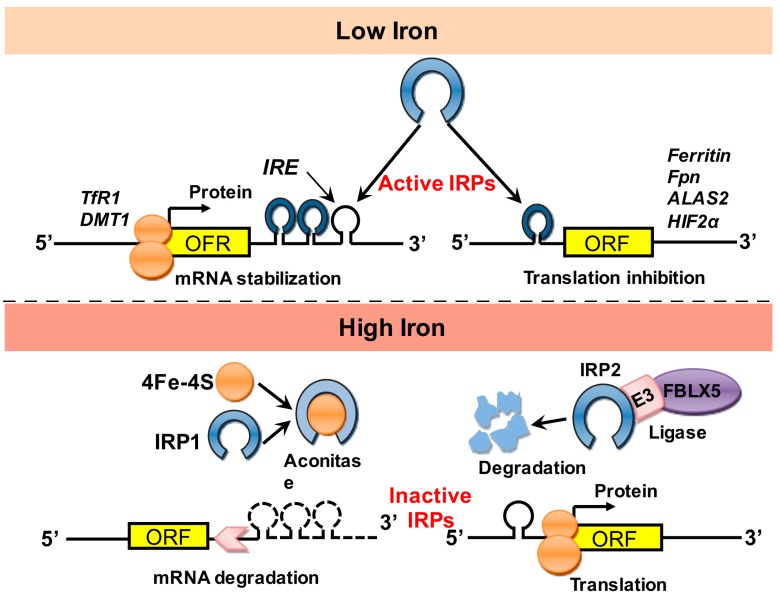
Post-transcriptional control of iron homeostasis. Iron-responsive element‒binding proteins (IRP1 and IRP2) bind to the iron-responsive element (IRE) in the untranslated region (UTR) of mRNAs encoding various iron-regulating molecules, thereby regulating their translation. ALAS2: aminolevulinic acid synthase 2; DMT1: divalent metal transporter 1; E3: ubiquitin ligase subunit; FBLX5: F-box and leucine-rich repeat protein 5; Fpn: ferroportin; HIF2α: hypoxia-inducible factor 2α; mRNA: messenger ribonucleic acid; ORF: open reading frame; TfR1: transferrin receptor 1.

In addition to transcriptional regulation, iron homeostasis can also be regulated at the translational level, representing an even more significant form of regulation. Accordingly, the iron-responsive element (IRE)‒binding proteins IRP1 and IRP2 bind to IREs in the untranslated regions (UTRs) of mRNAs of genes that encode a variety of iron-regulating molecules, including ferroportin, DMT1, and TfR1 [28] (Figure 3). For example, in an iron-deficient state, IRP2 binds to IREs in order to maximize cellular iron levels. Specifically, binding of IRP2 to IREs in the 5′-UTR of mRNAs encoding ferritin and ferroportin repress translation, while binding of IRP2 to IREs in the 3′-UTR of mRNAs encoding TfR1 and DMT1 stabilize the mRNA resulting in efficient translation.

## 2. Iron Toxicity, Oxidative Stress, and Antioxidants

### 2.1. Iron Toxicity

Iron is a potentially toxic molecule, as it can both donate and accept electrons. Iron can catalyze the formation of free radicals from reactive oxygen species (ROS) via the Fenton reaction, which is the reduction of H_2_O_2_ by a single electron to produce a hydroxyl radical [3,4,5]; this ultimately leads to damage to a wide variety of cellular structures. Therefore, the majority of iron is bound to other molecules for storage and/or transport, and only minute amounts of iron are available in the labile pool [16]. Even in this labile pool, however, iron is not completely unbound, as the majority is believed to form a complex with peptides, carboxylates, and/or phosphates [29,30,31,32]. Despite the abundance of complexes to which iron can bind in the body, iron levels in the body can occasionally exceed the pool of available transferrin molecules. For example, iron toxicity is common during iron overload states associated with genetic factors or acquired factors such as repeated blood transfusions. 

### 2.2. Oxidative Stress

Reactive species are formed as the result of normal metabolic processes. However, the human body is equipped with detoxifying mechanisms that regulate the generation of reactive species and can even repair damage caused by reactive species [33,34]. If radical species are not neutralized, they can damage proteins, lipids, nucleic acids, and other cellular components, serving as the underlying basis of many chronic diseases. Furthermore, oxidized cellular components can contribute to oxidative damage of other components and/or cause additional adverse oxidative changes [35]. Labile iron is the most important contributor of this oxidative damage to cellular components [3,4,5]. Superoxide radical (O_2_^−^) is the initial reactive species produced during these reactions, serving as the precursor for additional reactive radicals, including H_2_O_2_ and the hydroxyl radical (OH^−^), one of the most potent free radical species that can react with a wide range of cellular constituents [36]. 

#### 2.2.1. Oxidative Stress and Iron Deficiency Anemia

Iron deficiency can result from a variety of causes, including blood loss, nutritional deficiency due to inadequate intake of iron, and inhibition of iron absorption by certain foods and/or compounds such as calcium, phytates, tannins, and proton pump inhibitors. Diseases that affect the intestinal lining (for example, Crohn’s disease) can also affect iron absorption, leading to iron deficiency [37]. Iron deficiency first affects stored iron in the body; depletion of these stores is believed to produce non-specific symptoms such as dizziness, weakness and fatigue. Moreover, iron deficiency can have severe obstetric consequences for both the mother and fetus. In fact, iron deficiency anemia in infants and growing children has been associated with delayed psychomotor development and cognitive deficits due to abnormal neurodevelopment [38,39]. Oxidative stress does not necessarily result from iron deficiency, but it often appears as a co-morbidity, as the conditions that give rise to iron deficiency also promote oxidative stress. For example, during infection and/or inflammation, the body tends to absorb less iron in order to deprive the invading bacteria of the iron that they need to thrive [40]. Moreover, oxidative stress is a common feature in many chronic diseases with long-term iron deficiency; in this case, the negative regulation of ferroportin by hepcidin is believed to be responsible for the iron deficiency [26,27]. Additionally, the hypoxic condition induced by anemia may worsen oxidative stress via pro-oxidant changes, including altered cellular metabolism, increased catecholamine metabolism, and leukocyte activation, thereby leading to increased free radical production and oxidative stress [41]. In managing iron deficiency, iron supplementation can be given in order to build up iron stores for hemoglobin production; however, it is also important to manage any underlying conditions that could negate the benefits of iron supplementation therapy; in this respect, antioxidant therapy can ameliorate anemia-related oxidative damage [42]. 

#### 2.2.2. Oxidative Stress and Iron Overload

As discussed above, iron is potentially toxic due to the generation of free radicals via the Fenton reaction. Thus, the human body has developed processes to regulate the amount of iron absorbed in accordance with the body’s needs in order to prevent the adverse effects of iron overload. Despite these processes, however, iron overload can still occur. For example, ingesting large amounts of supplemental iron can damage the intestinal lining, causing increased absorption of iron into the body. Repeated blood transfusions and some genetic mutations are also associated with iron overload. For example, hereditary hemochromatosis (HH), which is caused by mutations in the genes that encode hemojuvelin (*HJV*, or *HFE2*) and hepcidin (*HAMP*) are associated with excessive iron absorption and iron-related toxicity, particularly in juvenile HH [43]. In general, the detrimental effects of iron overload begin to manifest when the complexes to which iron can bind become saturated. In particular, excess iron in the blood begins to deposit in tissues when available transferrin proteins are saturated. Large amounts of labile iron in the circulation can eventually damage the liver, heart, and other metabolically active organs [44]. Therefore, iron chelation is important for managing patients with iron overload, and returning iron levels to normal levels can help ameliorate the associated side effects.

#### 2.2.3. Iron Overload and Cell Death

Iron and ROS are important mediators of cell death in many organisms and in many pathological processes that involve altered iron homeostasis. Studies have shown that iron overload is associated with increased apoptosis, necrosis, and autophagy, albeit via different mechanisms [45,46,47,48]. Mitochondrial ROS are an important trigger of iron-induced apoptosis, which results in the peroxidation of cardiolipins and the release of cytochrome c to activate caspase-3/7 [48]. On the other hand, heme induces necrosis in macrophages via two synergistic mechanisms: (1) heme induces Toll-like receptor 4/Myeloid differentiation primary response gene 88- (TLR4/Myd88-) dependent tumor necrosis factor (TNF) expression; and (2) TNF activates the receptor-interacting protein (RIP) kinases 1 and 3, thereby initiating necrosis [47]. Finally, *Hamp* knockout mice fed a high-iron diet develop lysosomal iron overload in hepatocytes, leading to autophagy [46].

In addition to these “classic” mechanisms of cell death, an iron-dependent, oxidative form of cell death called ferroptosis has recently been identified. First described in Ras-mutated cancer cells treated with oncogenic Ras-selective lethal small molecules, ferroptosis is morphologically, biochemically, and genetically distinct from other forms of cell death, including apoptosis and necrosis [49,50]. The features of ferroptosis include an increased intracellular pool of liable iron, increased lipid peroxidation at the plasma membrane, and depletion of reduced nicotinamide adenine dinucleotide phosphate (NADPH); ferroptosis can be prevented by iron chelation or by the use of a lipophilic antioxidant, but not by inhibitors of other forms of cell death [49,50,51,52]. Furthermore, ferroptosis occurs in mouse models of hemochromatosis following iron overload [53]. Moreover, genetic and pharmacological inhibition of iron-related genes (e.g., *HO-1*, *TfR1*, and *FTH1*, which encode heme oxygenase-1, transferrin receptor 1, and ferritin, respectively) can inhibit ferroptosis in tumor cells [54,55,56]. Thus, although the relationship between ferroptosis and systemic iron metabolism is complex, it represents a promising target for managing iron-induced oxidative cell death, particularly in iron overload conditions [53]. 

### 2.3. Antioxidants

**Figure 4:**
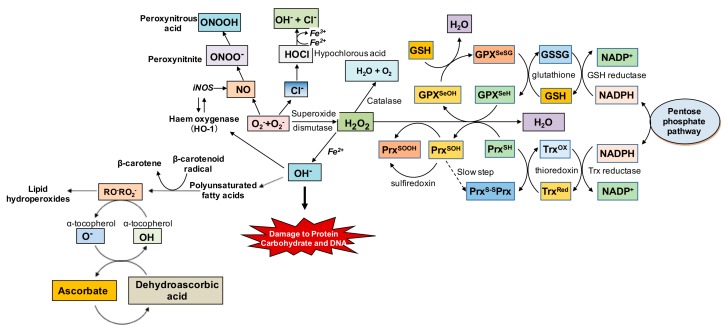
Summary of the oxidative stress cascade, including endogenous antioxidant defenses. Superoxide free radicals (O_2_^−^) generated during metabolic processes are endogenously neutralized by superoxide dismutase to hydrogen peroxide (H_2_O_2_), and subsequently to water (H_2_O) and oxygen (O_2_), although the glutathione (GSH) system can also neutralize H_2_O_2_ to H_2_O. Conversely, when O_2_^−^ is not neutralized, it can form more reactive species in the presence of nitric oxide (NO) and chloride (Cl^−^) thereby leading to further oxidative damage. Similarly, H_2_O_2_ in the presence of Fe^2+^ can produce hydroxyl free radicals (OH^−^), which are highly toxic to proteins and DNA, and can even lead to the generation of lipid peroxides that are also prooxidant. DNA: deoxyribonucleic acid; GSSG: glutathione disulfide; NADP: nicotinamide adenine dinucleotide phosphate; NADPH: reduced NADP; RO^−^: alkoxyl radical; RO_2_^−^: peroxyl radical.

The damage induced by oxidized cellular components is associated with the depletion of endogenous cellular antioxidant systems [33,34]. Numerous endogenous antioxidants maintain the cell’s redox state and prevent the harmful effects of oxidative stress; these antioxidants include superoxide dismutase (SOD), catalase, glutathione (GSH), thioredoxin (Trx), and ferritin (Figure 4). As discussed above, superoxide (O_2_^−^) is the first reactive radical produced, and this radical can be neutralized by SOD. There are three distinct SODs [57], each of which performs a specific function in human cells. SOD1 (Cu/Zn-SOD) is present in the cytoplasm, whereas SOD2 (Mn-SOD) is present in the mitochondria; SOD3, on the other hand, is almost exclusively extracellular [36].

When O_2_^−^ is neutralized, H_2_O_2_ is produced, which can be metabolized into non-toxic products by a catalase (H_2_O_2_ + H_2_O_2_ → 2H_2_O + O_2_) and glutathione peroxidase (GPx) in conjunction with glutathione (2GSH + H_2_O_2_ → GS–SG + 2H_2_O). Catalase is present in peroxisomes, whereas the location of the GPx depends on the subtype. For example, GPx1, GPx2, and GPx4 are cytoplasmic, whereas GPx3 is extracellular; GPx4 can also be found in the nucleus and endoplasmic reticulum [36]. Glutathione (GSH) is present in nearly all cells in the body and is present in high levels in organs with high oxygen consumption and energy production (e.g., the brain) [36,58]. In conjunction with its oxidized form (GSSG), GSH plays a major role in controlling cellular redox state. Similarly, the ubiquitous thioredoxin system also plays an important role in maintaining the cell’s redox state. Thioredoxins are small proteins with two adjacent cysteine residues that can undergo reversible oxidation to form a disulfide bond; when oxidized by H_2_O_2_ in a reaction catalyzed by Trx peroxidase in the presence of NADPH, this results in the generation of reduced Trx. The thioredoxin system protects the cell against oxidative stress—provided that sufficient NADPH is available. Thus, the production of Trx proteins and GSH increases under conditions of oxidative stress [36,59]. Finally, ferritin is considered an endogenous antioxidant, as it performs the important function of sequestering potentially toxic labile iron. 

**Figure 5:**
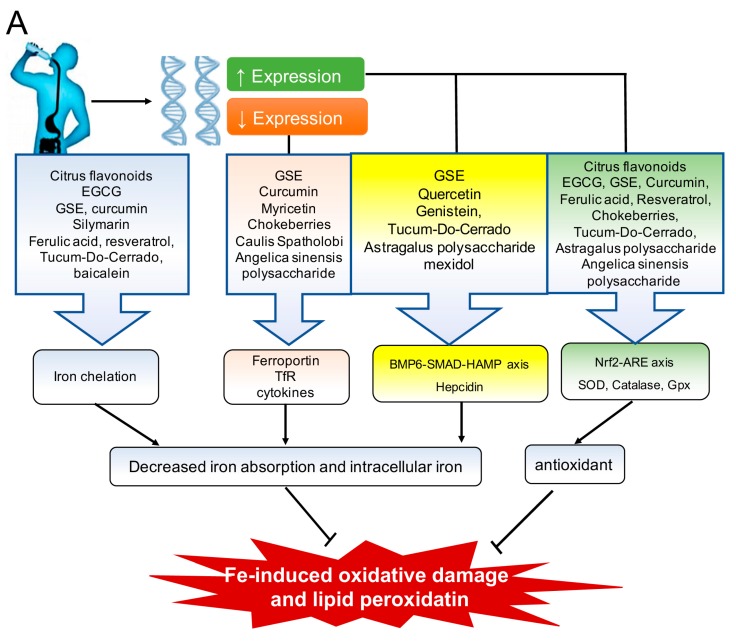

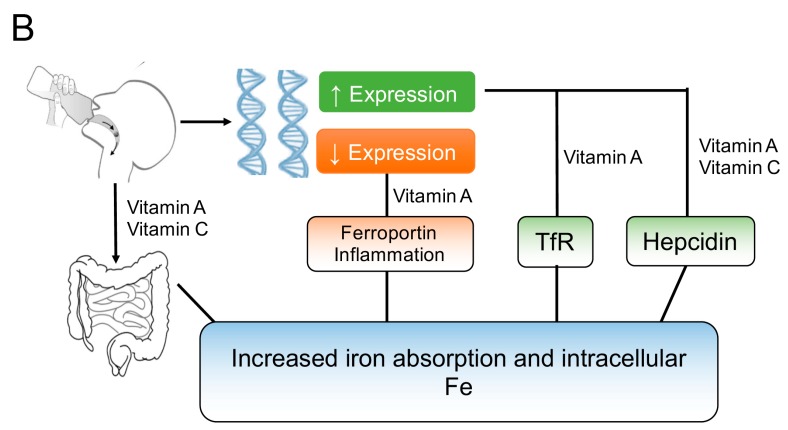
Overview of the mechanisms by which antioxidants regulate iron and oxidative stress. (**A**) A variety of antioxidants regulate iron absorption by chelating iron and/or by modulating the expression of antioxidant and iron metabolism‒regulating genes, ultimately reducing oxidative damage due to excess iron during iron overload; (**B**) Vitamins A and C can increase iron absorption and modulate the expression of several proteins (e.g., transferrin receptor (TfR), hepcidin, and ferroportin) and inflammatory signals, providing clinical benefits under iron deficient conditions. BMP6-SMAD-HAMP: bone morphogenetic factor-mothers against decapentaplegic homolog-hepcidin antimicrobial peptide; EGCG: epigallocatechin gallate; GPx: glutathione peroxidase; GSE: grape seed extract; Nrf2-ARE: nuclear factor erythroid 2-related factor 2-antixodant response element; SOD: superoxide dismutase.

Under certain conditions, endogenous antioxidants are unable to neutralize oxidative stress; in this case, exogenous antioxidants can be used to augment the body’s antioxidant systems. Thus, in addition to reducing the levels of oxidative stress common in many chronic diseases, antioxidants can also serve as an adjuvant to standard therapies in order to provide a synergistic clinical effect [60,61]. Vitamin A (beta-carotene), vitamin C, vitamin E (alpha-tocopherol), polyphenols, and other bioactive plant-derived compounds are effective exogenous antioxidants. Because several of these antioxidants can also regulate iron metabolism, they are ideal candidates for helping manage oxidative stress, particularly in the case of iron overload and/or iron deficiency (Table 1, Figure 5). Moreover, the addition of antioxidants to their respective therapies has been shown to provide superior effects against oxidative damage in iron overload [62,63] and iron deficiency anemia [42]. At the transcriptional level, antioxidant enzymes are regulated by the transcription factor Nrf2, which binds to the antioxidant response element (ARE) in the target gene’s promoter region. Nuclear factor erythroid 2-related factor 2 (Nrf2) is believed to be phosphorylated by protein kinase C (PKC), which causes the transcription factor to translocate to the nucleus, where it activates ARE-containing genes [36,64], ultimately leading to the neutralization of free radicals and the attenuation of oxidative damage. 

## 3. Bioactive Compounds That Regulate Oxidative Stress and Iron Metabolism

### 3.1. Polyphenols

Polyphenols are organic chemicals containing several phenol groups, which determine the chemical’s properties. Most polyphenols occur naturally as secondary metabolites in plants, where their primary role is to protect the plant from ultraviolet radiation and pathogens [90]. Interestingly, polyphenols can functionally counteract the effects of oxidative stress, making them suitable for therapeutic purposes. Moreover, polyphenols are reported to confer protection against the development of certain types of cancer, cardiovascular disease, diabetes, osteoporosis, and neurodegenerative disease [91]. Four main classes of polyphenols have been identified: flavonoids, phenolic acids, lignans, and stilbenes. 

#### 3.1.1. The Flavonoids: Tea Catechins, Black Soy Bean Seed Anthocyanins and Myricetin, Citrus Flavonoids, Grape Seed Extract, Curcumin, Quercetin, Genistein, and Silymarin

Flavonoids are the most common class of polyphenols and are present in a wide variety of plants, imparting many of these plants with their specific colors. Their primarily functions are to protect the plant and to act as chemical messengers, physiological regulators, and cell cycle inhibitors. However, because flavonoids are relatively non-toxic to animal cells, humans and other animals can safely ingest them, thereby benefiting from their positive properties [92]. Examples of flavonoids include quercetin, catechins, curcumin, and kaempferol, which are abundant in fruits, vegetables, legumes, red wine, and green tea. 

Table 1 summarizes the flavonoids and other antioxidants that regulate both iron homeostasis and redox state, in some cases via independent mechanisms. A flavonoid-rich extract of orange and bergamot juice has been shown to chelate iron in iron-overloaded A549 cells (a human lung epithelial cell line); in addition, this extract was shown to activate the antioxidant enzyme catalase, leading to a decrease in ROS production and membrane lipid peroxidation [65]. Based on these effects, the authors suggested that this flavonoid-rich extract is a promising candidate for regulating both iron homeostasis and oxidative stress [65]. Similarly, the tea-derived catechin epigallocatechin-3-gallate (EGCG) and grape seed extract (GSE)—both of which have potent antioxidant properties—are good candidates for chelating iron, given their ability to reduce basolateral iron export in Caco-2 cells [66]. Moreover, because EGCG can activate Nrf2—a master transcriptional regulator of antioxidant genes [67]—it may also help restore a balanced redox state in patients with iron overload. GSE is rich in anthocyanins, which are believed to be the bioactive compounds responsible for GSE’s ability to modulate iron homeostasis and oxidative stress [68]. Specifically, GSE-derived anthocyanins prevent sodium fluoride-induced oxidative damage in human embryo-derived hepatic cells by decreasing iron content and increasing antioxidants, including GPx, SOD, and total antioxidant capacity, by decreasing hepcidin expression and increasing ferroportin expression [68]. 

Another potent flavonoid antioxidant, curcumin, can also chelate iron in addition to modulating redox state [93]. Curcumin decreases iron levels in the bone marrow, spleen, and liver, and it can induce signs of iron deficiency anemia in mice by activating hepatic IRP and TfR1, as well as by repressing hepatic ferritin and hepcidin expression. Interestingly, curcumin can also reduce oxidative stress‒related inflammation induced by lipopolysaccharides (LPS) [69]. As discussed above, inflammation can activate hepcidin expression, which can reduce iron absorption, ultimately leading to iron deficiency. However, the fact that curcumin can reduce inflammation and hepcidin expression while inducing signs of iron deficiency suggests that this compound has iron chelation properties that are independent of hepcidin. 

Recently, Tang et al. [70] reported that quercetin can reduce hepatic iron deposition in mice that were exposed to either ethanol or excess iron. They found that quercetin increased BMP6, intranuclear SMAD4, SMAD4 binding to the *HAMP* promoter, and hepcidin expression, which led to decreased hepatic iron levels and reduced iron-related damage. Conversely, we reported that an anthocyanin-rich extract of black soybean seed coat extract can decrease hepatic hepcidin expression, resulting in decreased splenic iron and increased serum iron via reduced SMAD1/5/8 phosphorylation [71]. In a follow-up study, we found that myricetin is the principal bioactive compound in black soybean seed coat extract responsible for suppressing hepcidin expression [72]. Although myricetin is structurally similar to quercetin [94], unlike quercetin it inhibits hepcidin expression by modulating the BMP/SMAD signaling pathway in both in vitro and in vivo systems [72]. Specifically, myricetin decreases hepcidin expression by inhibiting the *HAMP* promoter; in addition, myricetin reduces SMAD1/5/8 phosphorylation in HepG2 cells, even in the presence of potent stimulators of hepcidin such as BMP6 and interleukin (IL)-6. In mice, myricetin also suppresses hepatic hepcidin expression, decreases splenic iron levels, and increases serum iron levels [72].

Genistein, another flavonoid that has potent antioxidant and anti-inflammatory properties, reduces ethanol-induced inflammation and oxidative stress in mice [95]. However, similar to quercetin, genistein increases *HAMP* promoter activity in both zebrafish and human hepatocytes via a Stat3- and Smad4-dependent process [73]. This suggests that genistein’s ability to induce hepcidin expression may be independent of its inflammation-reducing properties. Silymarin, another flavonoid, is present in milk thistle plant extract and may have iron-chelating properties [74]. Silymarin is actually a mixture of flavonolignans, including silibinin, isosilibinin, silicristin, and silidianin; silibinin has both antioxidant and hepatoprotective properties. Silymarin is a safe, well-tolerated, cost-effective alternative to currently available iron-chelation therapies for treating patients with β-thalassemia [74]. 

Flavonoids have already been shown to have both anti-inflammatory and antioxidant effects [96]. Recently, Bayele et al. reported that the transcription factor Nrf2 regulates hepcidin expression as part of an antioxidant regulatory network, whereas several flavonoids modulate iron homeostasis by blocking Keap1-mediated transcriptional repression, providing further evidence that Nrf2 modulates the activity of the *HAMP* promoter when activated as part of an antioxidant response [97]. However, the physicochemical properties of flavonoids can influence their pharmacokinetics, which can affect their bioavailability in vivo, thereby determining their ability to exert biological activities relevant to human health [98]. Thus, on one hand, flavonoids such as EGCG and curcumin can chelate iron, prevent the basolateral export of iron across Caco-2 cells, and induce signs of iron deficiency by increasing hepcidin expression and depleting iron stores, thereby providing clinical value during iron overload conditions and eventually restoring redox state; on the other hand, their iron-chelating property can also be detrimental and may even lead to iron deficiency. 

#### 3.1.2. Ferulic Acid

Ferulic acid is a phenolic compound present in a wide variety of plants. Ferulic acid can exist in the free form, or it can be bound to polysaccharides, flavonoids, fatty acids, and/or other phytochemicals, where it has antioxidant, anti-inflammatory, and other functional properties. The antioxidant effects of ferulic acid are believed to be mediated via the neutralization of free radicals [99]. Treating iron-overloaded mice with sodium ferulate reduces iron-induced oxidative stress, thereby reducing liver injury and apoptotic changes, increasing hepatic antioxidants, improving mitochondrial membrane potential, reversing mitochondrial swelling, and decreasing the production of ROS [75]. In contrast, unlike many flavonoids, ferulic acid does not affect iron uptake, suggesting that it does not have a direct iron-chelating property and that its effects are mediated by reducing the adverse effects induced by iron overload. 

#### 3.1.3. Resveratrol

Consuming moderate amounts of red wine has long been suggested to improve cardiovascular health, and this beneficial effect has been linked to resveratrol present in red wine [100]. The antioxidant effects of resveratrol may prevent adverse changes that lead to cardiovascular disease by modulating vascular cell function, low density lipoprotein (LDL) oxidation, and platelet aggregation, thereby reducing myocardial damage [100]. However, these ideas were recently called into question [101]. For example, resveratrol was recently reported to reduce iron-induced myocardial oxidative stress and myocardial fibrosis, ultimately reducing the risk of cardiomyopathy [76]. These effects appear to be mediated by attenuating iron-induced cardiac iron overload and oxidative stress, altering Ca^2+^ homeostasis, and reducing myocardial fibrosis. At the molecular level, resveratrol reverses iron-induced increases in cardiac nuclear and acetylated forkhead box protein O1 (FOXO1) levels, and it decreases the expression of both sirtuin 1 (SIRT1) and sarco/endoplasmic reticulum Ca^2+^-ATPase 2a (SERCA2a). 

#### 3.1.4. Chokeberries

Chokeberries can be eaten raw or in other food products, including wine, juice, and gummy chews. Chokeberries are rich in polyphenols, including anthocyanins, quercetin, and epicatechin [102,103], and the anti-inflammatory effects of chokeberry extract have been well-documented. For example, in an 8-week trial, athletes who consumed chokeberry juice had lower levels of inflammatory markers, as well as higher levels of total antioxidants and higher serum iron levels [77]. 

### 3.2. Vitamin A and Vitamin C

Both vitamin A and vitamin C have well-established antioxidant properties that are mediated via the attenuation of oxidative damage [104]. Vitamin A is a fat-soluble molecule that can interfere with the oxidation of polyunsaturated fatty acids in membrane phospholipids, thereby preventing lipid peroxidation [105]. Vitamin A metabolism also has implications with respect to iron homeostasis; indeed, vitamin A deficiency and iron deficiency are reported to co-occur in some populations [106]. Moreover, the serum levels of retinol (vitamin A1) are positively correlated with iron-related RBC indices, including serum iron, hemoglobin, and transferrin saturation levels [107,108]. In addition, vitamin A has been shown to affect the expression of transferrin receptors [109] and intestinal iron absorption [78]. Specifically, vitamin A modulates the expression of hepatic hepcidin and ferritin in mice, and it modulates ferroportin-1 (Fpn1) expression in Caco-2 cells via a hepcidin-independent mechanism, suggesting that several mechanisms may be involved in the vitamin A‒induced regulation of iron homeostasis, including changes in hepcidin-independent iron absorption and hepcidin-dependent iron mobilization [79]. In another study, beta-carotene—the precursor of vitamin A—was found to modulate iron absorption across Caco-2 cells even in the presence of the potent pro-inflammatory ligand IL-1β [80]. Specifically, beta-carotene: (1) attenuated iron-induced IL-8 release; (2) increased intracellular ferritin levels; and (3) reduced ferroportin levels. These changes resulted in normalized ferritin and ferroportin levels, reduced inflammatory signaling, and the release of intracellular trapped iron [80].

Vitamin C is a water-soluble molecule that can regenerate the radical form of alpha-tocopherol, thereby playing a major role in regulating redox state. In addition, vitamin C can influence intestinal iron absorption by affecting the reduction of Fe^3+^ to Fe^2+^ [1,2]. As discussed above, Fe^2+^ is imported into duodenal enterocytes via DMT1, and it exits the enterocytes through the basolateral membrane via Fpn1, which can be negatively regulated by hepcidin. Thereafter, transferrin transports the iron as Fe^3+^ to the bone marrow (for hematopoiesis), liver, and other organs (for storage). Aside from its role in iron reduction prior to intestinal absorption, vitamin C also regulates iron homeostasis by inhibiting hepcidin expression (for example, in HepG2 cells), potentially helping attenuate iron deficiency [81].

### 3.3. Other Plant Extracts

The desire to maximize the benefits of plant phytochemicals while avoiding the adverse effects often associated with synthetic pharmaceutical agents is fueling the search for new therapies based on plant-derived compounds [110]. In this regard, several plant extracts have been studied for their putative effects on iron homeostasis and oxidative stress, and plant phytochemicals present in the extracts of tucum-do-cerrado, astragalus, *Angelica sinensis*, *Caulis Spatholobi*, *Scutellaria baicalensis*, and others have shown promise.

Cerrado plant species are edible antioxidant-rich plants commonly found in Brazil. Among these species, the extract of the fruit of the tucum-do-cerrado plant (a fruit with a purple skin, whitish pulp, and a large seed) was shown to attenuate iron-induced increases in serum and tissue iron levels, transferrin saturation, and lipid oxidation (by increasing the expression of intestinal Nrf2 and hepatic hepcidin, ferritin, heme oxygenase 1 [Hmox1], NADPH dehydrogenase quinone 1 [Nqo1], Nrf2, and Bmp6), ultimately increasing the activity of antioxidant enzymes, including catalase, glutathione reductase, and GPx [82]. The authors found a general correlation between reduced oxidative damage and iron availability. Similarly, baicalein and baicalin are two major bioactive compounds with antioxidative properties found in the Chinese herb *Scutellaria baicalensis*. For example, in an in vitro assay, baicalein scavenged iron and inhibited the iron-mediated Fenton reaction under physiological conditions, thereby preventing oxidative damage [83]. Another Chinese herb, *Caulis Spatholobi*, is traditionally used to manage anemia. In Huh7 cells, this compound inhibits the expression of hepcidin, BMP6, and SMAD1/5/8; in mice, it reduces hepatic iron levels and increases serum iron levels by inhibiting hepcidin expression [84].

The polysaccharides contained in astragalus and *Angelica sinensis* have also been shown to modulate iron homeostasis and oxidative stress. These traditional Chinese herbs have been used for thousands of years for their anti-cancer and immunomodulatory properties. Recently, astragalus-derived polysaccharides were reported to suppress the accumulation of ROS and Nrf1 in human cardiac myocytes [85] and to increase hepcidin expression by activating the p38 mitogen-activated protein kinase (MAPK) signaling pathway and the release of IL-6 [86]. *Angelica sinensis* can protect chondrocytes from H_2_O_2_-induced oxidative stress by inducing changes in cell viability, SOD activity, catalase activity, malondialdehyde production, apoptosis, and inflammatory cytokines [87]. In addition, in mice transplanted with hepatoma-22 cells, *Angelica sinensis* reduces the serum levels of hepcidin, IL-6, ferritin, transferrin, TfR1, and TfR2 [88]. Emoxypine (trade name Mexidol), an antioxidant structurally similar to pyridoxine, was synthesized in Russia but is currently not used outside of Russia. Emoxypine is reported to have antioxidant and anti-inflammatory properties, and it has been shown to both reverse oxidative hemolysis and increase serum hepcidin levels in patients with hemochromatosis [89]. 

## 4. Conclusions

Many diseases and conditions related to a perturbation in iron homeostasis are associated with inflammation and oxidative stress, and a growing list of bioactive antioxidants and other plant-derived phytochemicals can simultaneously regulate iron homeostasis, oxidative stress, and inflammation (Figure 5). Some of these compounds are clinically beneficial with respect to lowering serum and/or tissue iron levels, whereas others may be beneficial to patients with iron deficiency. For example, with the exception of myricetin, most flavonoids reduce inflammation, chelate iron, and reduce iron absorption (and the resulting oxidative damage) predominantly via hepcidin-independent pathways; therefore, these compounds are beneficial in iron overload conditions. On the other hand, certain vitamins reduce inflammation and increase iron uptake and iron mobilization via both hepcidin-dependent and hepcidin-independent mechanisms, thereby providing clinical benefits in iron deficiency. Nevertheless, the majority of data collected to date are derived from in vitro and animal experiments. Therefore, further studies in humans are needed in order to evaluate the efficacy of these phytochemicals and their feasibility as a natural substitute for pharmaceutical agents, many of which are associated with adverse side effects. 

## Figures and Tables

**Table 1:** Select antioxidants and their mechanisms of iron regulation.

Antioxidant	Mechanism of Fe Regulation	Reference(s)
Citrus flavonoid-rich extracts of orange and bergamot juice	Chelation of iron in iron-overloaded human lung epithelial cells (A549), induction of catalase enzyme, and attenuation of reactive oxygen species (ROS) and membrane lipid peroxidation.	[65]
Epigallocatechin-3-gallate	Chelation of iron, reduced basolateral iron export in Caco-2 cells, and activation of nuclear factor erythroid 2-related factor 2 (Nrf2), a master transcriptional regulator of antioxidant genes in human mesenchymal stem cells (hMSCs).	[66,67]
Grape seed extract and anthocyanins	Grape seed extract induced chelation of iron and reduced basolateral iron export in Caco-2 cells. Anthocyanins induced attenuation of sodium fluoride-induced oxidative damage to human embryo hepatic cells via decreased iron content and increased antioxidants including glutathione peroxidase (GPx), superoxide dismutase (SOD), and total antioxidant capacity, mediated via decreased hepcidin and increased ferroportin expression.	[66,68]
Curcumin	Decreased iron levels in the bone marrow, spleen and liver, attenuated lipopolysaccharide (LPS)-induced oxidative stress-related inflammation, activated hepatic iron-responsive element-binding protein (IRP) and transferrin receptor 1 (TfR1), and repressed hepatic ferritin and hepcidin synthesis.	[69]
Quercetin	Attenuation of hepatic iron deposition in mice exposed to ethanol or excess iron, induction of bone morphogentic protein 6 (BMP6), intranuclear suppressor of mother of mothers against decapentaplegic homolog 4 (SMAD4), SMAD4 binding to hepcidin antimicrobial peptide (HAMP) promoter and hepcidin expression.	[70]
Black soybean seed coat anthocyanins	Reduced hepatic hepcidin expression, decreased splenic iron and increased serum iron, mediated via reduced SMAD1/5/8 phosphorylation.	[71]
Myrecitin	Reduced hepatic hepcidin expression, reduced hepcidin promoter activity, and reduced SMAD1/5/8 phosphorylation in HepG2 cells. Reduced hepatic hepcidin expression, decreased splenic iron levels, and increased serum iron levels in mice.	[72]
Genistein	Increased hepcidin expression and promoter activity in zebrafish and human hepatocytes in a signal transducer and activator of transcription 3- (STAT3-) dependent and SMAD4-dependent manner.	[73]
Silymarin	Iron chelation.	[74]
Ferulic acid	Attenuates iron-induced oxidative stress by reducing liver injury, apoptotic changes and ROS production; increases hepatic antioxidants and mitochondrial membrane potential; and reverses mitochondrial swelling.	[75]
Resveratrol	Attenuation of iron-induced cardiac iron overload, oxidative stress, altered Ca^2+^ homeostasis and myocardial fibrosis; increased cardiac nuclear and acetylated Forkhead box protein O1 (FOXO1) levels; Decreased sirtuin 1 (SIRT1) and sarco/endoplasmic reticulum Ca^2+^-ATPase 2a (SERCA2a) levels.	[76]
Chokeberries	Reduced inflammatory markers; increased total antioxidant status and serum iron levels.	[77]
Vitamin A and beta-carotene	Increased expression of TfR and hepcidin; increased intestinal iron absorption; reduced ferroportin expression; reduced inflammatory signaling; increased intracellular ferritin levels; release of intracellular trapped iron.	[78,79,80]
Vitamin C	Reduction of Fe^3+^ to Fe^2+^; inhibition of hepcidin expression.	[81]
Tucum-Do-Cerrado (Bactris setosaMart.)	Attenuation of iron-induced increases in serum and tissue iron levels, transferrin (Tf) saturation, and lipid oxidation via increasing expression of hepatic HAMP, ferritin, heme oxygenase 1 (Hmox1), NADPH dehydrogenase quinone 1 (Nqo1), and Nrf2 and BMP6, and intestinal Nrf2; increased antioxidant enzymes including catalase, glutathione reductase, and GPx.	[82]
Baicalein (Scutellaria baicalensis)	Iron chelation; inhibition of iron-mediated Fenton reaction under physiological conditions in vitro.	[83]
Caulis spatholobi	Inhibition of hepcidin, BMP6, and SMAD1/5/8 expression in Huh7 cells; reduced hepatic iron levels; increased serum iron levels in mice.	[84]
Astragalus polysaccharide	Attenuation of ROS and Nrf1 accumulation in human cardiac myocytes (HCMs); increased hepcidin expression via the activation of p38 mitogen-activated protein kinase (MAPK) and release of interleukin 6 (IL-6).	[85,86]
Angelica sinensis polysaccharide (ASP)	Increased chondrocyte cell viability, and increased SOD and catalase levels; reduced malondialdehyde production, apoptosis, and inflammatory cytokines; reduced levels of serum hepcidin, IL-6, ferritin, Tf, TfR1, and TfR2 in H22-bearing mice.	[87,88]
Mexidol	Reversal of oxidative hemolysis and increased serum hepcidin levels in hemochromatosis patients.	[89]
